# Vaccine Development for Herpes Simplex Viruses: A Commentary of Special Issue Editors

**DOI:** 10.3390/vaccines9020158

**Published:** 2021-02-16

**Authors:** Antonella Caputo, Peggy Marconi

**Affiliations:** Department of Chemical, Pharmaceutical and Agricultural Sciences, University of Ferrara, Via Fossato di Mortara 64/b, 44121 Ferrara, Italy

Herpes simplex virus type 1 and 2 (HSV1 and HSV2) are global, widespread human pathogens transmitted by direct contact that cause lifelong, recurrent asymptomatic and painful symptomatic clinical illnesses (cold sores, keratitis, blepharitis, meningitis, encephalitis, genital infections), overt disease and severe sequelae in neonatal and immune-compromised patients, and increased risk of cervical cancer and other sexually transmitted infections, including HIV. Moreover, repeated reactivation of latent HSV-1 in the brain is considered a major risk for Alzheimer’s disease (AD) pathogenesis [1,2,3,4,5,6,7,8,9]. Current drugs do not eradicate HSV latent infection and its reactivation, nor are they useful to treat asymptomatic infections which are unknowingly spread to new hosts. Drug resistance has also been reported at high levels in immunocompromised patients with severe clinical outcomes and death. To date, despite the major health burden caused by HSVs and 60 years of research, the correlates of protection are still unclear and there is currently no licensed preventative or therapeutic anti-HSV vaccine [10].

This Special Issue on “Vaccine development for Herpes Simplex viruses” includes reviews, research articles and brief reports focused on several strategies of vaccine development and immune responses, that are thought to be relevant for protection from infection and containment of virus reactivation.

Among the first type of contribution, Anthony Ike and coworkers [11] describe the interplay between innate and adaptive immune responses activated during HSV infections to control virus replication and the different immune evasion strategies developed by the virus to avoid clearance and favor the establishment of latent infection. The Authors also provide an interesting overview of the main prophylactic and therapeutic vaccination strategies that have been developed to date against HSV infections (mainly against HSV-2), including live-attenuated, replication-defective, subunit and DNA vaccines and their stage of development. Among extensive efforts for the development of prophylactic vaccines, several have primarily focused on generation of neutralizing antibodies against the viral envelope gD as the correlate of immune protection. Although promising preclinical results have been reported with these prophylactic vaccines, those that advanced to phase 3 clinical trial (i.e., the recombinant protein vaccine gD2 with AS04, MPL and alum adjuvants) have, unfortunately, failed. Another promising approach that has completed phase I clinical trials is a replication-defective HSV-2 strain deleted in two genes essential for virus replication (UL5 and UL29) (*dl*5-29) that generates neutralizing antibodies and low T cell responses. This review is, undoubtedly, important for the development of an effective vaccine against HSV.

A further interesting, different prophylactic strategy has been developed by Betsy Herold and coworkers [12] using a live-attenuated HSV-2 virus deleted in gD (∆gD-2). In this work, these researchers have analyzed the immune responses (IgG titers, neutralizing Ab and ADCC) elicited in mice by this vaccine as compared to those activated by the adjuvanted gD2 protein (gD2/AS04/MLP) and the *dl*5-29 replication-defective vaccines following different routes of administration (i.e., subcute, intramuscular, intradermal). The intradermal route of delivery proved more immunogenic for the gD subunit vaccine, whereas the intramuscular and intradermal routes induced similar responses and protection against challenge with a lethal dose of virus. Of interest, the ∆gD-2 viral vaccine induced the highest ADCC responses and the most effective protection against a lethal challenge and neurological latency. These results underscore the importance that the vaccine type and vaccine administration route have for inducing effective immune responses. In addition, the results indicate that an immune response to a broader spectrum of antigens rather than one single viral protein is likely to be more effective, especially when dealing with a complex pathogen such as HSV. Now, it will be interesting to verify if these promising results are confirmed in humans.

In line with these results, another enthralling strategy is reviewed by Richard Voellmy and coworkers [13]. They describe HSV-1 replication-competent-controlled viruses (RCCVs) for application to skin or mucosal membranes. These vaccines are characterized by stringent, deliberate spatial and temporal control of virus replication with increased safety and better protective immune responses than inactivated RCCVs and replication-defective HSV-1. The peculiarity of this strategy is that several rounds of RCCVs replication occur only in the presence of a specific activation. One example of such RCCVs is represented by recombinant HSV-1 RCCVs containing a replication-essential gene under the control of the human heat shock protein 70B (HSP70B) promoter, whose basal cellular activity is very low and is 1000-fold increased after heat activation above 42 °C as a consequence of the transient activation of the ubiquitous gene encoding heat shock transcription factor 1 (HSF1). Thus, a localized heat treatment at the site of injection activates vigorous replication of the RCCVs only transiently. This strategy combines the higher efficacy of the live-attenuated vaccines with the greater safety of the inactivated vaccines and its advantage is that expression of the regulated viral gene ceases shortly after the activation treatment. This interesting review highlights not only advantages and concerns of the RCCVs-based strategy but also its great potential for development of vaccines against heterologous antigens and other pathogens besides HSVs. In line with the concept that protective responses should be very broad both qualitatively and quantitatively and are still not fully clarified, are the results of the work of Roberta Mancuso and collaborators [14]. They describe a significant inverse correlation between HSV-1-specific IgG3 titers (the only IgG subclass that is not blocked by the HSV-1 Fc receptor and that counteracts viral humoral immunoevasion) and brain cortical thickness in specific areas involved in dementia and HSV-1 encephalitis in Alzheimer’s disease patients. The results suggest that an inefficient or decreased HSV-1-specific IgG3 humoral immune response may contribute to a progressive neurodegenerative process and shed light on an important correlate of protection for a vaccine therapeutic approach. The interesting contribution of Adalbert Krawczyk and coworkers [15] highlights instead the greater difficulty of HSV-2 compared to HSV-1 to be neutralized by monoclonal antibodies directed towards a gB epitope preserved in both viruses, and with equal affinity against the two HSV-1 and HSV-2 gB proteins, both in vitro and in the NOD/SCID mouse model. While the intravenous injection of these antibodies prolonged the survival of HSV-2-infected mice, it did not completely protect mice from death. Undoubtedly, these results should be taken into account for the development of both preventative and therapeutic approaches of vaccine strategies against HSV infections. Another observation relevant for the development of an anti-herpetic vaccine is described by Francesco Nicoli and coworkers [16] as they demonstrate the important role of the adjuvant for the maintenance of an efficient HSV-specific immune memory. They report that the treatment of mice, latently infected with HSV-1, with the HIV-1 Tat protein, which displays adjuvant functions, not only improves previously established HSV-specific memory responses but also prevents viral reactivation. IgG levels are directly correlated with the number of HSV-specific CD8+ T cells, suggesting an effect of Tat as an immune modulator on both arms of the adaptive immunity. Finally, Laith Aby-Raddad and coworkers [17] report the results of mathematical modeling analyses to assess in the US population the impact of even a partially efficacious prophylactic vaccine against HSV-2 (that reduces susceptibility to infection upon vaccination) and of a partially efficacious therapeutic post-exposure vaccine (that reduces HSV-2 genital lesions and the frequencies of recurrences). These interesting studies indicate that by 2050 even such a partially effective prophylactic vaccine could reduce annually the numbers of new infection by 58%, whereas such a partially effective therapeutic vaccine could reduce the transmission and the annual number of new infections by 12%. This contribution certainly provides important insights for development of an HSV-2 vaccination strategy, indicating that an effective vaccination strategy against HSV would offer a very cost-effective intervention to prevent the burden of genital herpes infections. This Special Issue collects multidisciplinary studies constituting a valuable source of knowledge for scientists working in the field of HSV vaccine development. In addition, it reveals the major challenges that persist in this field despite decades of ongoing efforts to develop a safe and efficacious HSV vaccine.
